# Supplementary material for: Differences in COVID-19-Related Hospitalization, Treatment, Complications, and Death by Race and Ethnicity and Area-Level Measures Among Individuals with Cancer in the ASCO Registry
Source: Cancers (Basel). 2025 Mar 2;17(5):857. doi: 10.3390/cancers17050857 (PMC11898501; doi:10.3390/cancers17050857)
Supplement: Supplementary file 1 [file cancers-17-00857-s001.zip › cancers-3461387-supplementary.pdf]

Table S1. Anti-COVID-19 Drug Use and COVID-19-Related Complications among Cancer Patients in the ASCO Registry from Confirmed SARS-CoV-2 Infection to End of the Acute Phase of Infection

COVID-19 CHARACTERISTICS	AT TIME OF CONFIRMED SARS-COV-2 INFECTION (T_0)[‡]							SHORT-TERM FOLLOW-UP PERIOD (T_1)[††]						
	Overall N (%)	AAPI N (%)	AIAN N (%)	Hispanic N (%)	NHB N (%)	NHW N (%)	*P*[†]	Overall N (%)	AAPI N (%)	AIAN N (%)	Hispanic N (%)	NHB N (%)	NHW N (%)	*P*[†]
**TOTAL**	5146 (100.0)	217 (4.2)	195 (3.8)	673 (13.1)	635 (12.3)	3426 (66.6)		3314 (100.0)	116 (3.5)	98 (3.0%)	465 (14.0)	395 (11.9)	2240 (67.6)	
**COVID-19 TREATMENTS**														
**Anti-COVID-19 drugs**							<.001							.01
No	2308 (44.9)	82 (37.8)	92 (47.2)	325 (48.3)	287 (45.2)	1522 (44.4)		1172 (35.4)	36 (31.0)	30 (30.6)	184 (39.6)	144 (36.5)	778 (34.7)	
Yes	1487 (28.9)	75 (34.6)	40 (20.5)	151 (22.4)	202 (31.8)	1019 (29.7)		531 (16.0)	15 (12.9)	14 (14.3)	55 (11.8)	56 (14.2)	391 (17.5)	
Remdesivir	609 (86.3)	28 (63.6)	21 (87.5)	71 (63.4)	72 (91.1)	417 (93.3)	.80	211 (6.4)	8 (6.9)	6 (6.1)	31 (6.7)	22 (5.6)	144 (6.4)	.97
Hydroxychloroquine	125 (50.8)	11 (35.5)	4 (57.1)	9 (15.0)	31 (73.8)	70 (66.0)	<.001	9 (0.3)	0 (0.0)	1 (1.0)	0 (0.0)	3 (0.8)	5 (0.2)	.11
Azithromycin	233 (66.2)	5 (20.0)	3 (60.0)	19 (27.1)	43 (79.6)	163 (82.3)	.002	68 (2.1)	1 (0.9)	2 (2.0)	7 (1.5)	11 (2.8)	47 (2.1)	.69
Convalescent plasma	169 (60.1)	7 (25.9)	6 (66.7)	23 (33.8)	15 (60.0)	118 (77.6)	.71	55 (1.7)	3 (2.6)	3 (3.1)	8 (1.7)	6 (1.5)	35 (1.6)	.57
Dexamethasone	642 (84.0)	25 (55.6)	17 (85.0)	58 (53.2)	81 (88.0)	461 (92.6)	.02	240 (7.2)	5 (4.3)	6 (6.1)	21 (4.5)	31 (7.8)	177 (7.9)	.08
Monoclonal antibodies	208 (19.9)	8 (17.8)	3 (7.3)	12 (8.6)	18 (10.0)	167 (26.1)	.002	107 (3.2)	1 (0.9)	1 (1.0)	7 (1.5)	9 (2.3)	89 (4.0)	.03
Other	495 (87.3)	31 (70.5)	11 (91.7)	51 (60.7)	74 (94.9)	328 (94.0)	.003	200 (6.0)	5 (4.3)	7 (7.1)	21 (4.5)	15 (3.8)	152 (6.8)	.08
Unsure or unknown	451 (8.8)	29 (13.4)	24 (12.3)	44 (6.5)	36 (5.7)	318 (9.3)		134 (4.0)	5 (4.3)	5 (5.1)	11 (2.4)	9 (2.3)	104 (4.6)	
**COVID-19-RELATED OUTCOMES**														
**Systemic complications**							<.001							.24
No	3864 (75.1)	126 (58.1)	143 (73.3)	461 (68.5)	466 (73.4)	2668 (77.9)		2668 (80.5)	94 (81.0)	78 (79.6)	372 (80.0)	310 (78.5)	1814 (81.0)	
Yes	418 (8.1)	36 (16.6)	11 (5.6)	64 (9.5)	64 (10.1)	243 (7.1)		153 (4.6)	7 (6.0)	3 (3.1)	30 (6.5)	20 (5.1)	93 (4.2)	
Bleeding	55 (6.0)	3 (4.9)	3 (7.1)	9 (5.6)	10 (8.6)	30 (5.6)	.17	34 (1.0)	1 (0.9)	2 (2.0)	7 (1.5)	6 (1.5)	18 (0.8)	.21
Disseminated intravascular coagulation	10 (1.2)	1 (1.8)	0 (0.0)	3 (1.9)	1 (1.0)	5 (1.0)	.24	5 (0.2)	1 (0.9)	0 (0.0)	2 (0.4)	0 (0.0)	2 (0.1)	.11
Sepsis	373 (30.9)	34 (39.1)	9 (19.1)	54 (27.1)	55 (34.8)	221 (30.8)	<.001	123 (3.7)	6 (5.2)	2 (2.0)	21 (4.5)	17 (4.3)	77 (3.4)	.53
Multiorgan failure[a]	NA	NA	NA	NA	NA	NA	NA	28 (0.8)	0 (0.0)	0 (0.0)	6 (1.3)	4 (1.0)	18 (0.8)	.71
**Pulmonary complications**							.16							.97
No	3531 (68.6)	123 (56.7)	134 (68.7)	449 (66.7)	418 (65.8)	2407 (70.3)		2486 (75.0)	89 (76.7)	73 (74.5)	359 (77.2)	286 (72.4)	1679 (75.0)	
Yes	798 (15.5)	37 (17.1)	25 (12.8)	87 (12.9)	109 (17.2)	540 (15.8)		337 (10.2)	12 (10.3)	9 (9.2)	45 (9.7)	42 (10.6)	229 (10.2)	
ARDS	174 (16.8)	10 (14.1)	1 (2.8)	21 (12.3)	32 (23.5)	110 (17.8)	.01	60 (1.8)	1 (0.9)	4 (4.1)	10 (2.2)	10 (2.5)	35 (1.6)	.18
Pneumonitis	234 (21.9)	9 (13.6)	4 (10.3)	23 (12.6)	39 (26.9)	159 (24.9)	.11	122 (3.7)	3 (2.6)	2 (2.0)	24 (5.2)	15 (3.8)	78 (3.5)	.47
Pulmonary embolism	78 (8.4)	1 (1.8)	4 (10.3)	8 (4.9)	14 (11.7)	51 (9.2)	.41	58 (1.8)	2 (1.7)	0 (0.0)	6 (1.3)	9 (2.3)	41 (1.8)	.59
Respiratory failure	605 (42.6)	28 (32.9)	21 (38.9)	68 (32.9)	80 (42.6)	408 (46.0)	.53	231 (7.0)	9 (7.8)	9 (9.2)	28 (6.0)	27 (6.8)	158 (7.1)	.77
**Cardiovascular complications**							.002							.05
No	4015 (78.0)	145 (66.8)	153 (78.5)	498 (74.0)	489 (77.0)	2730 (79.7)		2688 (81.1)	95 (81.9)	80 (81.6)	388 (83.4)	300 (75.9)	1825 (81.5)	
Yes	248 (4.8)	19 (8.8)	6 (3.1)	20 (3.0)	38 (6.0)	165 (4.8)		121 (3.7)	3 (2.6)	2 (2.0)	14 (3.0)	25 (6.3)	77 (3.4)	
Cardiac arrhythmia	142 (14.5)	10 (15.2)	3 (8.3)	4 (2.6)	20 (16.9)	105 (17.4)	.002	78 (2.4)	1 (0.9)	1 (1.0)	9 (1.9)	17 (4.3)	50 (2.2)	.09
Cerebrovascular accident	23 (2.7)	0 (0.0)	1 (2.9)	7 (4.4)	5 (4.7)	10 (2.0)	.03	13 (0.4)	1 (0.9)	0 (0.0)	1 (0.2)	3 (0.8)	8 (0.4)	.44
Congestive heart failure	57 (6.4)	4 (6.9)	1 (2.9)	5 (3.2)	9 (8.4)	38 (7.2)	.51	17 (0.5)	0 (0.0)	1 (1.0)	2 (0.4)	3 (0.8)	11 (0.5)	.65
Deep venous thrombosis	45 (5.1)	5 (8.3)	1 (2.8)	6 (3.8)	8 (7.1)	25 (4.8)	.07	22 (0.7)	1 (0.9)	0 (0.0)	2 (0.4)	5 (1.3)	14 (0.6)	.48
Myocardial infarction	15 (1.7)	2 (3.4)	0 (0.0)	2 (1.3)	1 (0.9)	10 (1.9)	.35	8 (0.2)	0 (0.0)	0 (0.0)	1 (0.2)	0 (0.0)	7 (0.3)	.91
**Other complications**							<.001							.10
No	3901 (75.8)	137 (63.1)	151 (77.4)	474 (70.4)	447 (70.4)	2692 (78.6)		2674 (80.7)	93 (80.2)	78 (79.6)	387 (83.2)	301 (76.2)	1815 (81.0)	
Yes	273 (5.3)	20 (9.2)	7 (3.6)	28 (4.2)	68 (10.7)	150 (4.4)		126 (3.8)	7 (6.0)	4 (4.1)	16 (3.4)	24 (6.1)	75 (3.3)	
Acute hepatic injury	49 (6.2)	7 (15.9)	0 (0.0)	6 (5.3)	5 (5.0)	31 (6.2)	.03	23 (0.7)	1 (0.9)	0 (0.0)	4 (0.9)	7 (1.8)	11 (0.5)	.07
Bowel perforation	3 (0.4)	0 (0.0)	0 (0.0)	0 (0.0)	2 (2.1)	1 (0.2)	.16	0 (0.0)	0 (0.0)	0 (0.0)	0 (0.0)	0 (0.0)	0 (0.0)	NA
Peritonitis	1 (0.1)	1 (2.6)	0 (0.0)	0 (0.0)	0 (0.0)	0 (0.0)	.08	2 (0.1)	0 (0.0)	0 (0.0)	1 (0.2)	1 (0.3)	0 (0.0)	.11
Acute renal failure	185 (20.4)	13 (24.5)	6 (16.7)	22 (17.9)	56 (38.1)	88 (16.1)	<.001	85 (2.6)	4 (3.4)	3 (3.1)	10 (2.2)	21 (5.3)	47 (2.1)	.006
Encephalopathy	83 (9.0)	2 (3.3)	1 (2.8)	4 (2.6)	14 (12.0)	62 (11.2)	.11	55 (1.7)	3 (2.6)	3 (3.1)	7 (1.5)	5 (1.3)	37 (1.7)	.57
Seizures	15 (2.0)	0 (0.0)	0 (0.0)	2 (1.9)	3 (3.1)	10 (2.1)	.88	6 (0.2)	1 (0.9)	0 (0.0)	0 (0.0)	0 (0.0)	5 (0.2)	.36

Abbreviations: AAPI, non-Hispanic Asian American and Pacific Islander; AIAN, non-Hispanic American Indian or Alaska Native; ARDS, acute respiratory distress syndrome; CVA, cerebrovascular accident; ICU, intensive care unit; IQR, interquartile range; NA, non-applicable; NHB, non-Hispanic Black; NHW, non-Hispanic White; *P*, P-value.

[†] For categorical variables, comparisons of proportions across race and ethnicity groups were assessed using the two-sided Chi-Squared test when <20% of expected cell counts were <5, and the Fisher's Exact Test when ≥20% of expected cell counts were <5. For continuous variables, comparisons of means across race and ethnicity groups were assessed using Analysis of Variance (ANOVA). The Benjamini-Hochberg (BH) approach was employed to correct for multiple comparisons, using a False Discovery Rate (FDR) of 0.05. Following correction, P-values ≤0.021 were statistically significant.

[‡] At *initial entry into the cohort (at confirmed SARS-CoV-2 infection)*, missing data for the overall study sample were >2% for the following categorical variables: *vaccination status* (31.0%); *patient received any care or treatment (for COVID-19 or cancer) via telemedicine* (7.8%); *patient received treatment for COVID-19 as part of therapeutic clinical trial* (9.1%); receipt of COVID-19 treatments such as *supplemental oxygen* (17.5%), *mechanical ventilation* (17.9%), *anti-COVID-19 drugs* (17.5%), and *other treatment approaches* (19.8%); COVID-19-related *hospitalization (*22.4%), *pneumonia* (25.7%), *systemic complications* (16.8%), *pulmonary complications* (15.9%), *cardiovascular complications* (17.2%), and *other complications* (18.9%). For continuous variables, the percentage of missing were as follows: *days on supplemental oxygen* (58.6%), days on *mechanical ventilation* (40.1%), days *hospitalized but not in ICU* (26.7%), *days hospitalized and in ICU* (44.0%), *days from diagnosis to COVID-19-related death* (0.5%).

[††] During the *short-term follow-up period (1-3 months post confirmed infection)*, missing data for the overall study sample were >2% for the following categorical variables: *vaccination status* (17.2%); *patient received treatment for COVID-19 as part of therapeutic clinical trial* (8.7%); receipt of COVID-19 treatments such as *supplemental oxygen* (44.6%), *mechanical ventilation* (44.6%), *anti-COVID-19 drugs* (44.6%), and *other treatment approaches* (44.6%); COVID-19-related *hospitalization (*44.5%), *pneumonia* (49.5%), *systemic complications* (14.9%), *pulmonary complications* (14.8%), *cardiovascular complications* (15.2%), and *other complications* (15.5%). For continuous variables, the percentage of missing were as follows: *days on supplemental oxygen* (58.8%), days on *mechanical ventilation* (20.8%), days *hospitalized but not in ICU* (17.3%), *days hospitalized and in ICU* (46.3%), *days from diagnosis to COVID-19-related death* (0.0%).

[a] Multiorgan failure was not evaluated when cancer patients first entered the cohort, *following a confirmed SARS-CoV-2 test*; only during the short-term follow-up period was this systemic complication assessed.

Table S2. COVID-19-Related Diagnosis, Treatment, Complications and Death among Cancer Patients in the ASCO Registry at the End of the Acute Phase of Infection, Overall and by Race and Ethnicity, N=3,314

COVID-19 CHARACTERISTICS	SHORT-TERM FOLLOW-UP PERIOD (T_1)[††]						
	**Overall N (%)**	**AAPI N (%)**	**AIAN N (%)**	**Hispanic N (%)**	**NHB N (%)**	**NHW N (%)**	***P***[†]
**TOTAL**	3314 (100.0)	116 (3.5)	98 (3.0%)	465 (14.0)	395 (11.9)	2240 (67.6)	
**Time of COVID-19 diagnosis**							<.001
Jan-Apr 2020	199 (6.0)	11 (9.5)	12 (12.2)	21 (4.5)	61 (15.4)	94 (4.2)	
May-Aug 2020	554 (16.7)	30 (25.9)	22 (22.4)	115 (24.7)	95 (24.1)	292 (13.0)	
Sep-Dec 2020	874 (26.4)	27 (23.3)	20 (20.4)	91 (19.6)	80 (20.3)	656 (29.3)	
Jan-Apr 2021	450 (13.6)	10 (8.6)	16 (16.3)	69 (14.8)	62 (15.7)	293 (13.1)	
May-Aug 2021	240 (7.2)	6 (5.2)	7 (7.1)	46 (9.9)	18 (4.6)	163 (7.3)	
Sep-Dec 2021	329 (9.9)	9 (7.8)	9 (9.2)	36 (7.7)	37 (9.4)	238 (10.6)	
Jan-Apr 2022	495 (14.9)	14 (12.1)	8 (8.2)	78 (16.8)	29 (7.3)	366 (16.3)	
May-Aug 2022	173 (5.2)	9 (7.8)	4 (4.1)	9 (1.9)	13 (3.3)	138 (6.2)	
**Vaccinated**							<.001
No	1115 (33.6)	31 (26.7)	24 (24.5)	170 (36.6)	151 (38.2)	739 (33.0)	
Yes	1074 (32.4)	30 (25.9)	25 (25.5)	153 (32.9)	81 (20.5)	785 (35.0)	
Unsure	555 (16.7)	20 (17.2)	26 (26.5)	50 (10.8)	56 (14.2)	403 (18.0)	
**Patient received any care or treatment (for COVID-19 or cancer) via telemedicine**							<.001
No	1978 (59.7)	59 (50.9)	51 (52.0)	225 (48.4)	217 (54.9)	1426 (63.7)	
Yes	956 (28.8)	44 (37.9)	30 (30.6)	207 (44.5)	137 (34.7)	538 (24.0)	
Unsure	356 (10.7)	10 (8.6)	17 (17.3)	31 (6.7)	34 (8.6)	264 (11.8)	
**Patient received COVID-19 treatment as part of therapeutic clinical trial**							.10
No	2996 (90.4)	101 (87.1)	84 (85.7)	434 (93.3)	345 (87.3)	2032 (90.7)	
Yes	31 (0.9)	3 (2.6)	0 (0.0)	8 (1.7)	2 (0.5)	18 (0.8)	
**COVID-19 TREATMENTS**							
**Hospitalization**							.48
No	1283 (38.7)	36 (31.0)	34 (34.7)	181 (38.9)	135 (34.2)	897 (40.0)	
Yes, but not in ICU	421 (12.7)	15 (12.9)	12 (12.2)	49 (10.5)	61 (15.4)	284 (12.7)	
Length of hospitalization (days), *median (IQR)*	7 (3-13)	4 (2-10)	8 (5-21)	7 (4-15)	8 (4-15)	6 (3-13)	.12
Yes, in ICU	134 (4.0)	5 (4.3)	3 (3.1)	20 (4.3)	13 (3.3)	93 (4.2)	
Length of ICU stay (days), *median (IQR)*	9 (3-15)	4 (2-6)	19 (11-26)	18 (5-29)	12 (8-12)	6 (3-15)	.03
**Receipt of supplemental oxygen**							.10
No	1294 (39.0)	40 (34.5)	32 (32.7)	195 (41.9)	155 (39.2)	872 (38.9)	
Yes	417 (12.6)	12 (10.3)	11 (11.2)	43 (9.2)	44 (11.1)	307 (13.7)	
Length of treatment (days), *median (IQR)*	7 (3-17)	6 (2-7)	11 (5-23)	5 (3-14)	5 (2-24)	7 (4-15)	.39
Unsure or unknown	126 (3.8)	4 (3.4)	6 (6.1)	12 (2.6)	10 (2.5)	94 (4.2)	
**Receipt of mechanical ventilation**							.63
No	1654 (49.9)	50 (43.1)	43 (43.9)	229 (49.2)	193 (48.9)	1139 (50.8)	
Yes	72 (2.2)	2 (1.7)	2 (2.0)	10 (2.2)	9 (2.3)	49 (2.2)	
Length of treatment (days), *median (IQR)*	10 (3-18)	10 (10-10)	36 (25-47)	17 (3-27)	9 (4-15)	9 (2-18)	.02
Unsure or unknown	111 (3.3)	4 (3.4)	4 (4.1)	11 (2.4)	7 (1.8)	85 (3.8)	
**Anti-COVID-19 drugs**							.01
No	1172 (35.4)	36 (31.0)	30 (30.6)	184 (39.6)	144 (36.5)	778 (34.7)	
Yes	531 (16.0)	15 (12.9)	14 (14.3)	55 (11.8)	56 (14.2)	391 (17.5)	
Unsure or unknown	134 (4.0)	5 (4.3)	5 (5.1)	11 (2.4)	9 (2.3)	104 (4.6)	
**Other treatment approaches**							.07
No	1511 (45.6)	45 (38.8)	40 (40.8)	220 (47.3)	168 (42.5)	1038 (46.3)	
Yes	164 (4.9)	5 (4.3)	4 (4.1)	16 (3.4)	30 (7.6)	109 (4.9)	
Unsure or unknown	162 (4.9)	6 (5.2)	5 (5.1)	14 (3.0)	11 (2.8)	126 (5.6)	

**COVID-19-RELATED COMPLICATIONS or DEATH**							
**Any complications**							.64
No	1016 (30.7)	35 (30.2)	25 (25.5)	151 (32.5)	108 (27.3)	697 (31.1)	
Yes	608 (18.3)	20 (17.2)	17 (17.3)	83 (17.8)	79 (20.0)	409 (18.3)	
**Pneumonia**							.67
No	1231 (37.1)	37 (31.9)	30 (30.6)	181 (38.9)	138 (34.9)	845 (37.7)	
Yes	444 (13.4)	14 (12.1)	14 (14.3)	54 (11.6)	53 (13.4)	309 (13.8)	
**Systemic complications[a]**							.24
No	2668 (80.5)	94 (81.0)	78 (79.6)	372 (80.0)	310 (78.5)	1814 (81.0)	
Yes	153 (4.6)	7 (6.0)	3 (3.1)	30 (6.5)	20 (5.1)	93 (4.2)	
**Pulmonary complications[a]**							.97
No	2486 (75.0)	89 (76.7)	73 (74.5)	359 (77.2)	286 (72.4)	1679 (75.0)	
Yes	337 (10.2)	12 (10.3)	9 (9.2)	45 (9.7)	42 (10.6)	229 (10.2)	
**Cardiovascular complications[a]**							.05
No	2688 (81.1)	95 (81.9)	80 (81.6)	388 (83.4)	300 (75.9)	1825 (81.5)	
Yes	121 (3.7)	3 (2.6)	2 (2.0)	14 (3.0)	25 (6.3)	77 (3.4)	
**Other complications[a]**							.10
No	2674 (80.7)	93 (80.2)	78 (79.6)	387 (83.2)	301 (76.2)	1815 (81.0)	
Yes	126 (3.8)	7 (6.0)	4 (4.1)	16 (3.4)	24 (6.1)	75 (3.3)	
**Death**							.94
No, living	2760 (83.3)	87 (75.0)	79 (80.6)	378 (81.3)	314 (79.5)	1902 (84.9)	
No, death due to cancer, complication of cancer treatment, other or unknown cause	180 (5.4)	7 (6.0)	5 (5.1)	19 (4.1)	24 (6.1)	125 (5.6)	
Yes, COVID-19 related death	117 (3.5)	3 (2.6)	4 (4.1)	13 (2.8)	14 (3.5)	83 (3.7)	
Length of time from diagnosis to death (days), *median (IQR)*	38 (18-80)	33 (30-111)	78 (11-110)	32 (22-81)	37 (17-78)	39 (18-80)	.56

Abbreviations: AAPI, non-Hispanic Asian American and Pacific Islander; AIAN, non-Hispanic American Indian or Alaska Native; ICU, intensive care unit; IQR, interquartile range; NHB, non-Hispanic Black; NHW, non-Hispanic White; *P*, P-value.

† For all categorical variables, comparisons of proportions across race and ethnicity groups were assessed using the two-sided Chi-Squared test. For continuous variables, comparisons of means across race and ethnicity groups were assessed using Analysis of Variance (ANOVA). The Benjamini-Hochberg (BH) approach was employed to correct for multiple comparisons, using a False Discovery Rate (FDR) of 0.05. Following correction, P-values ≤0.021 were statistically significant.

†† During the short-term follow-up period (1-3 months post confirmed infection), missing data for the overall study sample were >2% for the following categorical variables: vaccination status (17.2%); patient received treatment for COVID-19 as part of therapeutic clinical trial (8.7%); receipt of COVID-19 treatments such as hospitalization (44.5%), supplemental oxygen (44.6%), mechanical ventilation (44.6%), anti-COVID-19 drugs (44.6%), and other treatment approaches (44.6%); any COVID-19-related complications (51.0%), including pneumonia (49.5%), systemic complications (14.9%), pulmonary complications (14.8%), cardiovascular complications (15.2%), and other complications (15.5%). For continuous variables, the percentage of missing were as follows: days on supplemental oxygen (58.8%), days on mechanical ventilation (20.8%), days hospitalized but not in ICU (17.3%), days hospitalized and in ICU (46.3%), days from diagnosis to COVID-19-related death (0.0%).

[a] Systemic complications include bleeding, disseminated intravascular coagulation, sepsis, and multiorgan failure; pulmonary complications include acute respiratory distress syndrome, pneumonitis, pulmonary embolism, and respiratory failure; cardiovascular complications include cardiac arrhythmia, cerebrovascular accident, congestive heart failure, deep venous thrombosis, and myocardial infarction; and other complications include acute hepatic injury, bowel perforation, peritonitis, acute renal failure, encephalopathy, and seizures.
